# Portomesenteric Vein Thrombosis, Bowel Gangrene, and Bilateral Pulmonary Artery Embolism Two Weeks after Laparoscopic Sleeve Gastrectomy

**DOI:** 10.1155/2015/705610

**Published:** 2015-10-21

**Authors:** David G. Darcy, Ali H. Charafeddine, Jenny Choi, Diego Camacho

**Affiliations:** Department of Surgery, Montefiore Medical Center, New York, NY 10467, USA

## Abstract

Sleeve gastrectomy and gastric bypass surgery are popular and effective options for weight loss surgery. Portomesenteric vein thrombosis (PMVT) is a documented but rare complication of bariatric surgery. Proper surgical technique, careful postoperative prophylaxis, and early mobilization are essential to prevent this event. The diagnosis of PMVT in the postoperative period requires a high index of suspicion and early directed intervention to prevent a possibly fatal outcome. We present a case of PMVT complicated by small bowel ischemia resulting in gangrene that necessitated resection.

## 1. Introduction

Sleeve gastrectomy has recently become a very popular weight loss surgery [1] and portomesenteric vein thrombosis (PMVT) is a rare but documented complication after this operation [2]. The diagnosis can be challenging, and early intervention is key in the management of these patients. The case we are reporting is a PMVT complicated by small bowel gangrene that necessitated resection and primary anastomosis. Further workup for hypercoagulable state was negative; a high index of suspicion for venous thrombosis in the surgical bariatric patient must be maintained at all times, including several weeks after operation.

## 2. Case Presentation

Our patient is a 55-year-old female with a body mass index (BMI) of 41.1, with multiple medical problems including coronary artery disease, diabetes mellitus, hypertension, and hyperlipidemia. She failed nonsurgical attempts at weight loss with diet and exercise and elected to undergo a laparoscopic sleeve gastrectomy (LSG).

The patient is placed on the operating room table in supine position with sequential compression devices (SCD) on both lower extremities. Subcutaneous heparin is given preoperatively as well as 2 grams of cefoxitin thirty minutes before incision time. The surgeon stands on the right side of the patient and the assistant on the left. The abdomen is insufflated with CO2 to achieve pneumoperitoneum at 15 mm Hg. A LigaSure device (Covidien, Dublin, Ireland) is used to dissect the greater curvature of the stomach starting 5 cm from the pylorus. A 34F bougie was introduced and placed along the lesser curvature. Gastric resection is performed with Tri-Staple (Covidien, Dublin, Ireland) devices using black and purple loads, directed towards the angle of His. The resected stomach is finally extracted by extending a 12 mm trocar incision in the supraumbilical midline by 2-3 cm. Operative time was 53 minutes.

In the postoperative period, the patient remained on subcutaneous heparin as chemical prophylaxis against deep vein thrombosis, and her SCD were on for the entire hospital stay. She tolerated a clear liquid diet on her first postoperative day and was ambulating with minimal pain. She was sent home on post-operative day (POD) #2. She was seen in the clinic a week after discharge and was doing well, free of pain, and tolerating her diet.

**Figure 1:**
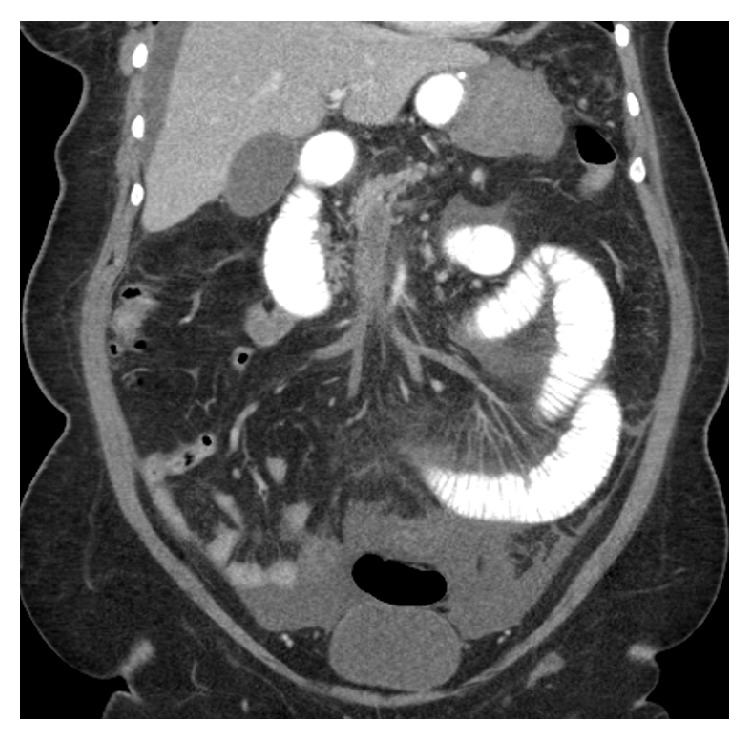
Coronal view with PO and IV contrast showing large portal venous thrombosis and dilated loops of small bowel in the left lower quadrant.

**Figure 2:**
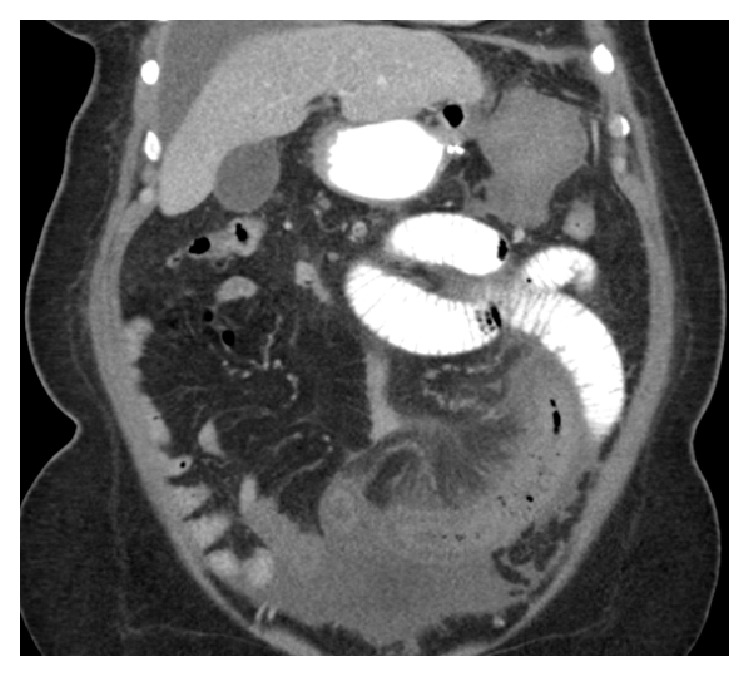
Coronal view with PO and IV contrast showing dilated loops of small bowel with wall thickening and pneumatosis denoting ischemia.

On POD #14 the patient presented to the ED with acute onset abdominal pain, nausea, and vomiting (nonbloody, nonbilious). Her vital signs were within normal limits, and physical exam was significant for a soft abdomen, which was nondistended, with tenderness in the right upper quadrant. An abdominal ultrasound showed thickened small bowel loops. Her labs were significant for leukocytosis and acidosis, and her heart rate increased as she was in the emergency department. A decision was made to obtain a CT of the abdomen and pelvis with PO and IV contrast for further assessment. The CT showed extensive superior mesenteric vein thrombosis (Figure 1), nonocclusive thrombi within the splenic vein, right common iliac vein, and its external and internal iliac branches. Additionally, there were concerns for ischemia/necrosis over a long segment of small bowel (Figure 2).

A heparin drip was started immediately to treat the PMVT and the patient consented to an emergent exploratory laparotomy. On exploratory laparotomy, gangrenous bowel in the mid-jejunum was found and resected (43 cm) and bowel continuity was achieved with primary stapled anastomosis. The sleeve gastrectomy staple line was examined and was intact.

The patient tolerated the surgery well; however she went into respiratory failure requiring intubation on POD #2. Despite being on a heparin drip at a therapeutic level, she developed bilateral pulmonary artery emboli. She improved clinically, and an IVC filter was placed on POD #5; she was extubated on POD #6, was started on the bariatric diet on POD #8 and warfarin on POD #9, and was eventually discharged home in good condition on warfarin. Her factor V Leiden and thrombophilia tests all came back negative. The patient returned to clinic 1 week after discharge; she was doing well, tolerating a bariatric diet without pain. She has been seen in hematology clinic at 1, 3, and 6 months after the readmission, doing well on warfarin. Hematology has suggested that she remain on lifelong anticoagulation for persistent portal thrombosis.

## 3. Discussion

The incidence of PMVT after LSG is reported at 1% [3], and following Roux-en-Y gastric bypass, the incidence is even lower [4]. The etiology of PMVT after LSG is still not clear. The classic Virchow triad explains that endothelial damage, stasis of venous blood, and a hypercoagulable state are the reasons behind formation of a thrombus. It has been suggested that induction of pneumoperitoneum is associated with decreased splanchnic circulation contributing to the formation of a clot; however, this operation does not require a higher pressure than others [5]. Morbid obesity and those patients with metabolic syndrome are at increased risk for clot formation, and morbidly obese patients are thought to have a baseline inflammatory state, further contributing to the formation of blood clots [6, 7]. It is our practice to send patients with BMI >60 home on prophylactic enoxaparin for two weeks, and they are seen in clinic at that time.

Although placement of IVC filter was considered early on, the patient was managed with heparin drip after her bowel resection and PMVT, given that filters themselves are thrombogenic. Deep venous thrombosis alone is not an indication for IVC filter placement when therapeutic heparin drip can be employed. The development of bilateral pulmonary embolism on treatment with therapeutic heparin prompted IVC filter placement.

Patients with PMVT after surgery present with a wide variety of symptoms [8]. Our patient had tenderness in the right upper quadrant and symptoms that could have been mistaken for acute cholecystitis, but her overall clinical picture was more consistent with bowel ischemia. As in our case, PMVT can lead to catastrophic outcomes including necrotic bowel, septic shock, and death. Although arterial insufficiency is a more common cause of bowel ischemia, the gangrene that developed in this patient was due to impaired venous outflow. Therefore, early diagnosis and intervention (anticoagulation and prompt surgical exploration if needed) are critical in saving this subset of patients with a highly morbid and possibly fatal complication.
